# Supplementary material for: Classification of naturally evoked compound action potentials in peripheral nerve spatiotemporal recordings
Source: Sci Rep. 2019 Jul 31;9:11145. doi: 10.1038/s41598-019-47450-8 (PMC6668407; doi:10.1038/s41598-019-47450-8)
Supplement: Supplementary file 1 — Supplementary Tables [file 41598_2019_47450_MOESM1_ESM.pdf]

Supplementary Information for (SREP-18-43299A)**:**

Manuscript Title: Classification of naturally evoked compound action potentials in peripheral nerve spatiotemporal recordings

Authors: Ryan G. L. Koh, Adrian I. Nachman, and José Zariffa

Supplementary Table 1

Supplementary Table I. Pairwise comparisons on the mean F1-scores with Bonferroni Correction for the 3-class problem

**Algorithm**	Random Forest	Neural Network	RF-RBI 1 ms	RF-RBI 10 ms	RF-RBI 33 ms	RF-RBI 100 ms
Matched Filter	**0.02045**	**0.01023**	0.29667	**0.00235**	**0.00011**	$\mathbf{4 \times 10^{-8}}$
Random Forest	-	0.25182	1	**0.01376**	**0.00034**	$\mathbf{7.3 \times 10^{-6}}$
Neural Network	-	-	1	**0.02913**	**0.00058**	$\mathbf{3.3 \times 10^{-5}}$
RF-RBI 1 ms	-	-	-	**0.00011**	$\mathbf{5.9 \times 10^{-6}}$	$\mathbf{1.5 \times 10^{-5}}$
RF-RBI 10 ms	-	-	-	-	**0.03382**	0.10320
RF-RBI 33 ms	-	-	-	-	-	0.45633
RF-RBI 100 ms	-	-	-	-	-	-

Significant values have been bolded

Supplementary Table 2

Table II. Pairwise comparisons on the mean F1-scores with Bonferroni Correction for the 2-class problem

**Algorithm**	Random Forest	Neural Network	RF-RBI 1 ms	RF-RBI 10 ms	RF-RBI 33 ms	RF-RBI 100 ms
Matched Filter	0.18010	0.07480	0.38958	**0.00232**	**0.00038**	$\mathbf{4.1 \times 10^{-7}}$
Random Forest	-	1	1	**0.01234**	**0.00132**	$\mathbf{9.1 \times 10^{-5}}$
Neural Network	-	-	1	**0.00444**	**0.00037**	$\mathbf{1.9 \times 10^{-5}}$
RF-RBI 1 ms	-	-	-	**0.00454**	**0.00075**	**0.00164**
RF-RBI 10 ms	-	-	-	-	0.07667	0.14654
RF-RBI 33 ms	-	-	-	-	-	1
RF-RBI 100 ms	-	-	-	-	-	-

Significant values have been bolded
